# Pullulan for Advanced Sustainable Body- and Skin-Contact Applications

**DOI:** 10.3390/jfb11010020

**Published:** 2020-03-18

**Authors:** Maria-Beatrice Coltelli, Serena Danti, Karen De Clerk, Andrea Lazzeri, Pierfrancesco Morganti

**Affiliations:** 1 Department of Civil and Industrial Engineering, University of Pisa, 56122 Pisa, Italy; serena.danti@unipi.it (S.D.); andrea.lazzeri@unipi.it (A.L.); 2 Consorzio Interuniversitario Nazionale per la Scienza e Tecnologia dei Materiali (INSTM), 50121 Florence, Italy; 3 Department of Materials, Textiles and Chemical Engineering, Faculty of Engineering and Architecture, Ghent University, Technologiepark 70A, 9052 Ghent, Belgium; Karen.DeClerck@ugent.be; 4 Department of Mental Health and Physics and Preventive Medicine, Unit of Dermatology, University of Campania “Luigi Vanvitelli”, 80138 Naples, Italy; 5 Academy of History of Health Care Art, 00193 Rome, Italy

**Keywords:** pullulan, biopolymers, exopolysaccharides, biodegradation, biocompatibility

## Abstract

The present review had the aim of describing the methodologies of synthesis and properties of biobased pullulan, a microbial polysaccharide investigated in the last decade because of its interesting potentialities in several applications. After describing the implications of pullulan in nano-technology, biodegradation, compatibility with body and skin, and sustainability, the current applications of pullulan are described, with the aim of assessing the potentialities of this biopolymer in the biomedical, personal care, and cosmetic sector, especially in applications in contact with skin.

## 1. Introduction

**Figure 1:**
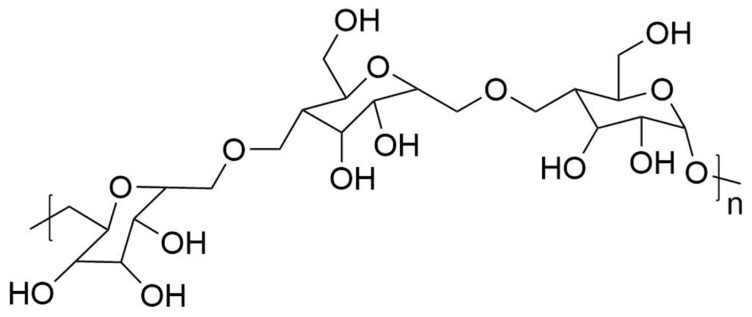
Structure of pullulan.

Pullulan (PUL) is a biopolymer produced by strains of the polymorphic fungus *Aureobasidium pullulans* as an extracellular, water soluble polysaccharide [1,2,3]. This natural fibrous polymer, which is produced using various substrates such as starch, distilled by-products, bakery waste, or agro-industrial residues is a linear, unbranched, odorless, tasteless, and neutral exopolysaccharide resistant to changes in temperature and pH. PUL [4], produced from the fungus as an elongated branched septate and large chlamydospores during its life-cycle, consists of α-(1,6)-repeated maltotriose units via an α-(1,4)glycosidic bond with chemical formula (C_6_H_10_O_5_)_n_ and the structure reported in Figure 1.

The alpha linkages give PUL physical properties such as water solubility and fiber flexibility, enabling its capacity of forming fibers and films, which is similar to certain petrol-derived plastics [5]. However, it is a non-ionic, non-hygroscopic, non-immunogenic, non-toxic, non-carcinogenic, non-mutagenic polymer that can be processed into a transparent and edible film, thanks to its excellent filming and casting properties [6,7]. Moreover PUL was declared safe with a Generally Recognized as Safe (GRAS) status by the Food and Drug Administration in USA and certified harmless for food usage by regulations in many countries. Thus, it has potential industrial applications in food, pharmaceutical, cosmetic, and biomedical fields [8] and many reviews have been recently dedicated to its production and applications [9,10,11,12,13,14]. In fact, this polymer is a flocculant, foaming, and adhesive agent and for its antioxidant and prebiotic properties, it is used in low calorie dietary fibers in healthy and functional food [6,7]. Moreover, PUL also permits cross linking and delivery of genetic material and some therapeutic cytokines, thanks to its fibrous characteristic and chemical structure in each repeating unit multiple –OH groups, suitable for scaffolds compatible with physiological conditions [15].

Thus, the structure results show that it is ideal for the hydrogel-based delivery of both cells and biomolecules, due also its own physicochemical property, which exhibits water retention capability. If used on the submicron-to-nanoscale dimension or electrospun, PUL fibers possess several advantages: huge surface area to volume ratios, accessibility and flexibility in surface modifications as well as excellent mechanical properties such as tensile strength and modulus, suggesting a huge potential for biomedical, cosmetic, and engineering applications [6].

Thus, PUL electrospun fibers used in combination with other natural-origin biomaterials contribute to make a tissue as structured as the natural extracellular matrix (ECM). Therefore, PUL shows good capacity to support and regulate the cell types involved in skin homeostasis [16]. Indeed, ECM provides a microenvironment that allows for nutrient diffusion and modulates the biochemical and physical stimuli among the different cells, inducing their favorable interactions, increasing the proliferation rate, maintaining their phenotype, supporting the differentiation of stem cells, and activating cell-signaling [17].

On one hand, biomaterials and their relative combinations (e.g., polymers and composites in their different forms: non-woven tissues, films, emulsions) enable support and correct structural architecture for tissue regeneration, ultimately improving quality of life, when used in medical and cosmetic applications [18]. In addition, scaffolding materials play a critical role in tissue engineering by serving as an artificial ECM to provide temporary mechanical support for the cells and mimic their natural nanofibrous environment [19]. Additionally, thanks to the electrospinning technique, the PUL fibers can be used to produce non-woven tissues and films to be further physically coated or chemically bound by various active ingredients. These ingredients may help to produce advanced medications and innovative cosmetic products as well as coatings for medical, food, and cosmetic packaging [20,21]. However, the non-toxicity and easy biodegradability of this natural compound enables the production of innovative biopolymer-based nanocomposites, both skin-friendly and environmentally friendly. Indeed, these products, based on renewable biowaste sources, may be utilized as scaffolds for medical, food, and cosmetic use [22,23]. Therefore, for the above-mentioned interesting characteristics, PUL is highly useful to fabricate innovative cosmetic products free of preservatives and other chemicals, which are often the cause of allergic and sensitizing phenomena. These safe and smart cosmeceuticals could modulate the aging phenomena as they are positively involved in the orchestra of signaling, regulating the reproduction and survival of different skin cells. Moreover, being made from agroforestry waste, this new category of biomedical/personal care/cosmetic products could help to reduce the CO_2_ production and save the environment, ultimately preserving natural raw materials and biodiversity of our planet for the incoming generations [18,19].

## 2. Polysaccharides and Waste

Over the last decades, many natural biopolymers have shown to be of interest for many different industrial fields including pharmaceutical, biomedical, and cosmetics. Among them, the extracellular polysaccharides (EPS), obtainable from agri-food waste such as the fungal polysaccharides including glucans, mannans, and chitinous polymers are considered interesting. Thus, bioconversion of the waste by-products using biotechnological ways offers two important advantages: (a) a reduction in environmental pollution by sustainable development ways; and (b) to provide value-added biomaterials with a wide spectrum of activities in many industrial sectors such as pharmaceutical, food, cosmetics, and other industrial sectors [24]. These biomaterials can be classified into several categories: neutral (beta-glucan, dextrans, cellulose, and pullulan); acid (alginic acid, hyaluronic acid); basic (chitin, chitosan), or sulfated (heparin sulfate and chondroitin sulfate). However, there is a need to obtain biomaterials by cost-effective processes that are able to ensure the total utilization of processing wastes through green eco-friendly technologies. Among all of these biomolecules, polysaccharides are the more inexpensive and easily degradable bio-products, representing, by ~75%, the majority of all organic materials on earth [25]. They are, therefore, the most abundant natural polymers on the planet, serving as major element in plants (i.e., cellulose) and animals (i.e., chitin in arthropods and hyaluronic acid in mammals) or as a food storage mechanism (i.e., starch or glycogen). These biopolymers, which include plant exudates such as gum arabic, Karaya gum, and tragacanthin gum; seed gums such as guar gum; and algal or microbial polysaccharides such as xanthan gum and pullulan are the simplest building blocks of natural carbohydrate molecules that comprise repeating units joined together by glycosidic bonds, are often used as chemical markers, particularly in cell recognition [26,27]. They are found, therefore, as components of glycoproteins and glycolipids showing, among others, immunostimulation and antioxidant effects. However, apart from the natural raw material found in nature from which it is possible to obtain polysaccharides, a great quantity of them can be extracted from agri-food waste, accounting for about 300 billion tons/year [28,29]. By utilizing this waste, it will be possible to preserve natural materials for the incoming generations, eliminating part of the pollutants and reducing the production of greenhouse gas (GHG) emissions.

## 3. Exopolysaccharide Synthesis and Applications

As previously reported, PUL is an extracellular, linear, unbranched, and water-soluble microbial polysaccharide that is produced by microorganism fermentation similar to dextran and xanthan gum. It belongs to EPS and shows film forming properties [3]. It is interesting to underline that EPS, synthesized from microorganisms to produce the biofilm, are responsible for the physicochemical and biological properties of this microbial structure, which involves, among others, surface adhesion, cell–cell communication, protective barrier, and water retention as well as protective activity against predation by protozoa, oxidizing biocides, UV, and environmental stress. For their interesting properties, these EPSs can be used in many industrial applications. Thus, the different EPS are used in the pharmaceutical and cosmetic industry as bio-polymeric carriers for controlled release and in tissue engineering as well as in agriculture to improve the fertility and productivity of soil, or in the food industry to produce lactic acid and in the wastewater for color removal [30,31]. Naturally, the production of exopolysaccharides depends on the type of microorganism, the system, and substrate adopted with the operating condition and the biomass utilized. However, substrate composition and environmental conditions seem to be the main factors affecting the production of EPS [32]; especially if there exists no single set of culture that may assure their high productivity due to the different requirements requested from microorganisms such as temperature, pH, aeration rates, and fermentation conditions [33]. Thus, these and other biodegradable polysaccharides have been used and continue to be used in many different medical and cosmetic applications, opening new opportunities in future delivery technology.

## 4. Pullulan Characteristics, Applications, and Market

PUL is one of the emerging biopolymers synthesized and elaborated by the polymorphic fungus *A. pullulans*, characterized by its non-hygroscopic nature, and water-solubility, showing a relatively low viscosity compared to other polysaccharides [34]. Similar to chitin, it possesses good thermal stability, showing decomposition temperatures in the range of 250–280 °C. PUL can be produced by utilizing the waste generated by many agri-food as the substrate. In fact, they are rich in carbon and nitrogen sources and other nutrients, so they are essential for the adequate growth of *A. pullulans*. However, although the large-scale production of this polymer has been developed, the major problem remains its discoloration from the pigment melanin present in the fermentation broth of the fungus. Although it has the facility to be processed into a transparent and edible film with interesting gas barrier and anti-static properties, the obtained film becomes sticky by absorbing atmospheric moisture at 80% relative humidity (RH) [35]. Therefore, the necessity to blend and laminate it with other polymers occurs such as agar, alginate, carrageenan, or other polymers. Interestingly, PUL shows good adhesive and binder properties, is non-irritant, non-toxic, edible, biodegradable, skin- and environment-friendly, thus having properties that are extremely useful in different industrial applications, as previously reported [34,36]. Thus PUL, being resistant to mammalian amylases and providing low calories, is used to produce low-calorie dietary products as starch replacers and functions as a prebiotic to promote the growth of beneficial bifidum bacteria. Moreover, its adhesive properties may be used as a binder and stabilizer in food pastes, denture adhesive, and tobacco and as an adhesive to stick nuts to cookies. Additionally, for its water retention and film forming properties, it may result in an interesting moisturizing and protective ingredient for cosmetic lotions, powders, facial packs, and protective agent for hair shampoos, hair dressings, and tooth powders [20,34,35,36,37,38,39,40]. PUL, in fact, is produced by microorganisms because they like to surround themselves with a highly hydrated EPS layer to protect their organisms from desiccation and against predation by protozoa [33,37]. Additionally, it is interesting to underline that fungal polysaccharides such as PUL have shown to stimulate the immune response, have antimicrobial activity, and lowers cholesterol and triglycerides. Moreover, it can develop peculiar extended structures including hydrogels or micro/nanoparticles that may be used as biomaterials for other specific medical and cosmetic applications [3]. Pullulan has also been used in composite materials for biomedical applications (e.g., bone substitutes) [41,42,43,44].

**Figure 2:**
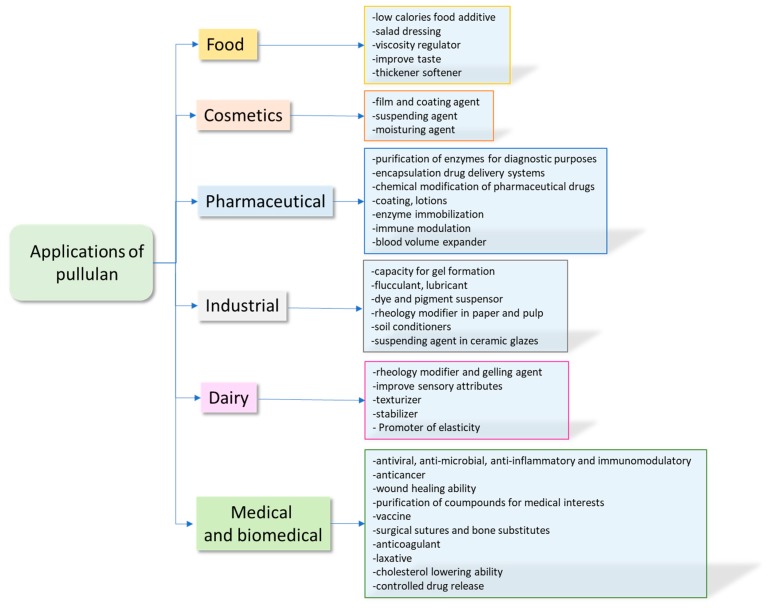
Applications of pullulan.

The applications of pullulan are shown in a schematic in Figure 2. However, it must be pointed out that many research groups are continuing their investigations into its interesting properties. Hence, it is reasonable that in the future, the number of applications will grow extensively. 

Regarding its physicochemical characteristics, the molecular weight of PUL ranges from 362 to 480 kDa and is highly influenced by: (a) types of strain used; (b) initial medium pH; (c) nature and composition of substrate used; (d) media composition; and (e) time of harvesting [36]. Regarding the productive process, its highest molecular weight is obtained by the fermentation of agri-food waste by different steps, whereas that obtained from a synthetic medium such as glucose generally have a lower molecular weight. Thus, the microbial fermentation of PUL is carried out in five steps: (a) the harvesting of microbes; (b) removal of undesired by-products such as melanin and cellular proteins, indispensable for its purity; (c) precipitation of polysaccharide; (d) ultracentrifugation/dialysis; and (e) freeze drying. Sometimes, its purity can be further improved by the use of ultrafiltration membranes [36]. For this purpose, agro-based industries such as potato, sugar, rice, grape, and coconut processing generate a huge amount of solid/liquid waste that can cause severe environmental issues, if not discarded [25]. However, aeration, the appropriate pH range between 5.5 and 7.5, the presence of specific enzymes, glucose, and a nitrogen source are considered fundamental for optimizing both fermentation and yield in pullulan [20,36]. Moreover, different mathematical models can be used to facilitate the control and optimization of pullulan production while several analysis techniques are available during and after the fermentation period to control its purity, especially when used for medical and cosmetic purposes. In conclusion, while PUL—compared to the other exopolysaccharides—shows a high potentiality of applications for its interesting bioactive properties, the production cost represents the critical challenge for its wide diffusion in the market. Hence, the costs must be further reduced to become competitive with other microbially-produced polysaccharides [20]. Thus, the pullulan market is expected to grow at a Compound Annual Growth Rate (CAGR) of roughly 2.2% over the next five years and will reach US$ 130 million in 2023 from US$ 129 million in 2017 [37]. Currently, the pharmaceutical industry accounted for the largest markets with about 40.74% of the global consumption in 2015, showing Japan as the biggest market with 667 million tons, China ranked second with a share of 20.65%, and the USA was third with a share of 29.65% [37].

However, in our opinion, this sugar-like polymer could have a greater future in the medical and cosmetic sectors, if innovative carriers for new and smart products are realized in the coming years [38,39,40,41].

## 5. Production, Biodegradation, and Biomedical Applications of Pullulan

PUL is the major exopolysaccharide synthesized intracellularly by the polymorphic fungus *A. pullulans* and further secreted out to the cell surface. First isolated and characterized by Bernier (1958) from culture broths of *A. pullulans*, PUL has become the object of an ever increasing research effort. So far, different microbial sources of PUL have been found: *Tremella mesenterica, Cytaria harioti, Cytaria darwinii, Cryphonectria parasitica, Teloschistes flavicans,* and *Rhodototula bacarum;* however, *A. Pullulans* is still the main microorganism used to produce PUL [33]. Elemental analysis revealed PUL to have the chemical formula C_6_H_10_O_5_. Treatment with the extracellular enzyme pullulanase, derived from *Enterobacter aerogenes*, led to maltotriose subunits as the main product. Infrared spectroscopy proved the presence of both α and β-glycoside bonds along the backbone of the polysaccharide and the co-existence of α-(1→4)- and α-(1→6)-glycosidic linkages in the PUL structure [33]. To obtain PUL, the microorganism is cultured in a fermenter, and different fermentation parameters have been used [20] Glucose units needed the presence of three key enzymes to be converted into pullulan, namely: α-glucose mutase, uridine diphosphoglucose pyrophosphorylase (UDPG) pyrophosphorylase, and glucosyltransferase. Aside from glucose, *A. pullulans* also consumes sucrose, mannose, galactose, maltose, fructose, and even agricultural wastes; the presence of hexokinase and isomerase are necessary for *A. Pullulans* to convert different carbon sources into PUL. It can be synthesized by cell-free enzymes of *A. Pullulans* when both UDPG and adenosine triphosphate (ATP) are present in the reaction mixture. Indeed, ATP is essential for biosynthesis, and UDPG cannot be replaced by ADPG [45]. Isotopic labelling experiments revealed that lipid-linked oligosaccharides were produced during PUL biosynthesis. Since 1976, Hayashibara Company Limited (Okama, Japan) has been the main commercial producer of PUL. A continuum fermentation process, with acidic pH, nitrogen, and carbon sources is used to obtain PUL. The main problems related to PUL production are a decrease in molecular weight, the high viscosity of the fermentation broth, and the discoloration of the polysaccharide resulting from the simultaneous synthesis of melanin pigment.

Polymer degradation occurs through scission of the main chains or side chains of its macromolecules. There are different factors that can induce polymer degradation such as thermal activation, hydrolysis, biological activity (due to enzymes action), oxidation, photolysis, and radiolysis [46]. Polymer biodegradation is thus a consequence of its chemical and physical conditions and interaction with the environment it is in contact with, which can be the human body (e.g., implanted device) or water or soil, if the manufact is disposed of. Biodegradation can occur due to biological activity, in which microorganisms identify the polymers as a source of organic building blocks and a source of energy they need for life. Environmental factors both regulate the polymer to be degraded and have an impact on the microbial population, therefore parameters such as humidity, temperature, pH, and others are really important in the biodegradation process. PUL can thus be degraded by enzymatic action [44]. These enzymes can be divided into four groups depending on what bond they act on: (1) Pullulanase enzymes perform hydrolysis on the α(1→6) glycosidic bonds of pullulan, leading to the formation of maltotriose; (2) Isopullulanase hydrolyzes the α(1→4) glycosidic bonds of pullulan, leading to isopanose; (3) Neopullulanase hydrolyzes the α(1→4) glycosidic bonds of pullulan, leading to panose; and (4) glucoamylase enzymes, which lead to the hydrolysis of pullulan, giving glucose as the major degradation product. Among these groups, we can find the α-amylase enzyme, which is mainly secreted by salivary glands and the pancreas and acts on the α(1→4)/α(1→6) glycosidic bonds [44]. There are other enzymes that are able to degrade the pullulan, but are not found in the human body such as β-amylase. PUL degradation follows this reaction:

Biodegradable polymer (PUL) + Microorganisms/Enzymes → CO_2_ + H_2_O + Biomass

Reaction products (the biomass) are biocompatible, therefore the immune system is able to interact with them. Moreover, a material such as PUL complies with second (biodegradable/bioactive) and third generation (induce tissue regeneration and function) biomaterials, thanks to its biocompatibility. After its approval by the FDA, PUL and its many derivatives are widely exploited in the food industry, pharma and cosmetic products, and biomedical applications including tissue engineering [47,48,49,50]. Pullulan derivatives are obtained by reactions of esterification, sulfation, oxidation, and others, or by the grafting of chemical structures to the main backbone. For example, PUL/nano-hydroxyapatite composites proposed for bone tissue engineering have shown potential to regenerate bone defects both in vivo and in vitro [42]. 

Methacrylated pullulan (PULMA) hydrogels were printed via multiscale light-assisted 3D printing techniques by using visible stereolithography apparatus (SL) and two-photon lithography (TPL), thus enabling 3D patterns from the millimeter down to the micron range. Mechanical properties, in particular rigidity, were controlled by adding a bifunctional crosslinker. PULMA structures were cytocompatible with mesenchymal stem cells, finally confirming the ability of this polysaccharide to be used in the biomedical field [51] 

PUL and its derivatives such as nanogels, nanoparticles, and microspheres, can act as efficient carriers in drug delivery systems (DDS), reducing the toxicity of drugs and improving their activity and stability.

Moreover, the nano/micro size ensures the persistence in blood circulation of DDS for a prolonged time so that they can achieve their therapeutic role. Some examples include tumor, gene, and liver targeting. The efficiency of antitumor drugs like doxorubicin (which is toxic for the heart and stomach) is enhanced using a PUL-based system that is able to target cancer cells. PUL-based DDS for anticancer drugs include pH-sensitive PUL nanoparticles [52]. Usually, the PUL is modified to have a spacer acting as an acid-sensitive bond that is stable at physiological pH, but hydrolysable under acidic conditions, in order to conjugate the drug to the PUL backbone. To obtain gene targeting, cationized PUL (mixed in aqueous solution rich of Zn^2+^ with plasmid DNA) was shown to favor drug release; the PUL-PEI (low Mw polyethyleneimine) conjugate supports non-viral systems due to the high DNA affinity and blood compatibility. Finally, high affinity between liver exosomes and cationized PUL, which easily accumulates in the hepatic tissue, improves the activity of its specific macrophages to better suppress the inflammation.

## 6. Pullulan and Nanotechnology

Nanotechnology aims at understanding and exploiting the techniques used by nature to make its ingredients, which range in the nano domain. It consists of the construction and characterization of nanoscale materials and structures exhibiting enhanced physicochemical properties compared to the bulk materials. A nanometer, in fact, is one billionth of a meter and typical atoms are about one third of a nanometer. On the other hand, the so called bionanotechnology is focused on the ways the nanotechnology is used to make devices, bio-macromolecules, and products that are able to copy and deliver biological machines that are similar to nature, which are necessary to regenerate biological tissues [53]. Transmucosal delivery is the first-line option for the systemic delivery of many drugs and nanoparticles have been generally demonstrated to improve protein pharmacokinetic profiles, not only providing increased stabilization, but also allowing for controlled release and enhanced drug absorption. Pullulan-based nanoparticles have been reported to adhere to the nasal epithelium in a study regarding nasal vaccination [54], and the adherence of pullulan nanoparticles to respiratory epithelial cells, although to a limited extent, was verified [55].

Hydrophobic derivatives of pullulan, namely cholesteryl-pullulan, form nanoparticles by self-aggregation. These nanoparticles have been demonstrated to form stable complexes with both hydrophobic and hydrophilic drugs such as proteins, reinforcing their flexibility [56]. Reversibly disulfide-crosslinked pullulan nanoparticles with folic acid decoration were fabricated for dual-targeted and reduction-responsive anti-tumor drug delivery due to the specific affinity of pullulan and folic acid to overexpressed ASGPR (asialoglycoprotein receptor) and FR (folate receptor) on the surface of tumor cells, respectively [57]. Phthalyl pullulan nanoparticle (PPN)-treated *Lactobacillus plantarum* was synthesized and characterized to develop a new type of prebiotic for *Lactobacillus plantarum* [58]. These polymeric nanoparticles, as prebiotics, can exert substantial effects on probiotics, which lead to the increased production of an antimicrobial peptide that is powerful against Gram-positive and Gram-negative pathogens.

The interactions at the interfaces between nanomaterials and biological systems, therefore, is of significant interest in discovering the material activity at the nano level. Nanoparticles, in fact, interact with small organic ligands, therapeutic molecules, proteins, DNA, and cell membranes, establishing a series of nanoparticle/biological interfaces that depend on colloidal forces as well as dynamic bio-physicochemical interactions [59]. Thus, it is possible to establish a direct connection and functionality between the biomolecules of the skin-cell structures and the nanoparticles and nanocomposites produced and used, depending on their composition, size, shape, surface charge, and physicochemical functions. These interactions could impart unique physical properties to the nanomaterials, simultaneously regulating the biological responses of the bio-nanoconjugates [59]. However, it should be underlined that all nanomaterials have been distinguished and are acting in dependence of their natural origin, and are incidental and engineered nanoparticles by which they are made. It should also be remembered that the efficiency of a specific controlled-release of any nano-system is determined by its physicochemical properties and biodegradation rate [60]. Controlling and mimicking nano-biomolecules and nano devices, therefore, represents the greatest and fundamental challenge of nanotechnology. This nano dimension may be obtained, for example, by the electrospinning technology as reported below. The goal of the medical and cosmetic sectors in tissue engineering is to use biomaterials to produce bioengineered scaffolds and cell therapies to act as “smart band aids” to replace senescent and/or diseased resident cells, reestablishing their anatomy and physiology [61]. A better knowledge of the longer-term effectiveness and safeness of these novel materials is important in order to realize innovative drugs for preventing and trying to solve many diseases in regenerative medicine as well as to formulate smart cosmetic products that are able to prevent and slow down the signs of aging and photo-aging. The production of nanostructured tissues is possible thanks to electrospinning techniques. More specifically, electrospun PUL could be used to improve tissue engineering scaffolds by increasing their cytocompatibility by acting on the surface nano-rugosity and nano-porosity of these materials. Its biofunctionality and biocompatibility, in fact, may help to produce tissues that are able to mimic the native extracellular matrix (ECM) [62]. To improve the final product strength and further tune the properties, our group has worked for some years on the use of nanochitin, nanolignin, and its complexes [20,21,22,23,24,38,39,40,41,53]. Multiscale structures of PULMA enabled the control of mechanical properties by the virtue of cross-linking [51]. Moreover, different nanoparticles can be obtained by this polysaccharide and its derivatives [63].

## 7. Pullulan and Extra Cellular Matrix

Thus, PUL and other polymers seem able to provide not only support, but also the correct structural architecture essential for tissue regeneration [18]. Moreover, a combination of different polymers such as chitin nanoparticles and nanolignin and their complexes that are used to deliver selected active ingredients can enhance the efficacy of the final formulation [38,39,40,41]. They can, in fact, play a critical role, serving as a synthetic ECM for cells, mimicking the native microenvironment that lead to tissue formation. These biopolymers, therefore, allow nutrient diffusion, also modulating the biochemical and physical stimuli necessary to guide cell proliferation, differentiation, migration, and growth [64]. Nutrition, reproduction, and communications are the three vital functions of living beings. Cells communicate with each other and with connective tissues via signals that are sent by surface molecules or membrane proteins. Thus, it seems possible that PUL effectiveness, especially when the polymer is nanostructured, could be connected to a better and gradual comprehension of the finely orchestrated nature of intercellular communication, enhancing the innate surveillance mechanism of the skin and the continuous turnover of its cells, altered, for example, during premature aging. Thus, as previously reported, there is the necessity to formulate products through a biomimetic approach; adopting problem-solving methods inspired by nature’s functions to make structures from the molecular level as nature does. An interesting technique is the electrospinning method to make non-woven tissue through the use of biopolymers. Through this technology, it is possible to produce scaffolds that are able to directly influence cell viability, migration, proliferation, and differentiation, thanks to their specific surface and structural properties [64]. Electrospinning is therefore a particularly promising cost-efficient technique for tissue engineering to fabricate biomimetic nano matrices, characterized by their surface to volume ratio and interconnecting pores, which is indispensable for cell adhesion and survival. This technique, useful to produce continuous ultra-thin, nano-scaled, non-woven dry fibers collected as non-woven tissues, involves the use of a high-voltage electrostatic field applied to create electrically charged jets of polymer solutions [65,66]. This stems from the principle in which a liquid droplet could be charged by electrostatic charge to form a fiber. In its setup, therefore, there are three basic components: the material extrusion system equipped with microscale spinneret, high-voltage power supply, and counter electrode for collecting fibers. Thus, electrospinning is a highly versatile technique that allows for the processing of different biopolymers into nanofibers that are covered or bound by various nanoparticles, and embedded by selected active ingredients. Through this technology, it is possible to produce a great variety of non-woven tissues, which having a particular morphology mimicking the native ECM and containing proper ingredients, that may be used in the medical and cosmetic field. Naturally, the formation and structure of the fibers is dependent on their chain entanglements and concentration as well as on the chemical structure of the polymers used. Compact globular-like polymer chains produce fewer entanglements than random walk-coil chains at the same concentration [66]. The concentration of the polymer solution—used as the main parameter to control the fiber diameter—is critical and needs to be controlled in order to obtain spun fibers, and also because it modulates viscosity and surface tension of the final solution. Regarding PUL manufacturing, it has already been electrospun with success from an aqueous solution, and parameter mapping including environmental parameters such as humidity and temperature allows for reproducible electrospun mats with tuned fiber diameters [67]. Sun et al. [68] obtained pullulan nanofibers with a diameter of 100–700 nm using redistilled water as the solvent through electrospinning technology. The result was achieved by the control of the processing parameters such as polymer concentration, applied voltage, distance between the capillary and collector, and flow rate. Karim et al. [69] prepared electrospun mats based on pullulan/montmorillonite clay nanocomposites. The study showed that the introduction of clay resulted in the improvement in tensile strength and thermal stability of the PUL matrix. The coexistence of intercalated montmorillonite layers and an increase in the crystallinity of the blended nanofiber mats with the addition of clay filler were also observed.

Interestingly, Qian et al. [70] incorporated up to 7.41% of rutin as a UVA and UVB absorber in electrospun pullulan/poly(vinyl alcohol). Rutin (3′,4′,5,7-tetrahydroxyflavone-3b-D-rutinoside) is one of the most abundant flavonoids from natural sources and has many biological properties, being anti-inflammatory, antiallergenic, and antimicrobial. Wang and Ziegler [71] prepared electrospun nanofiber mats from aqueous starch-pullulan dispersions. Moreover, Wang et al. [72,73] prepared electrospun gelatin/PUL fibers mimicking ECM. Hence, PUL was electrospun in combination with specific polymer, nanofillers, or compounds for obtaining functional nanostructured mats. In fact, as previously reported, among the several features that can affect a scaffold’s performance are fiber surface properties such as roughness, topography, and chemistry as well as porosity, pore size, inter connectivity, mechanical properties, and degradation rates of the non-woven-tissues, aside from the atmospheric conditions (mainly the humidity and the temperature of the environment).

## 8. Skin Structure and Skin Contact Applications 

**Figure 3:**
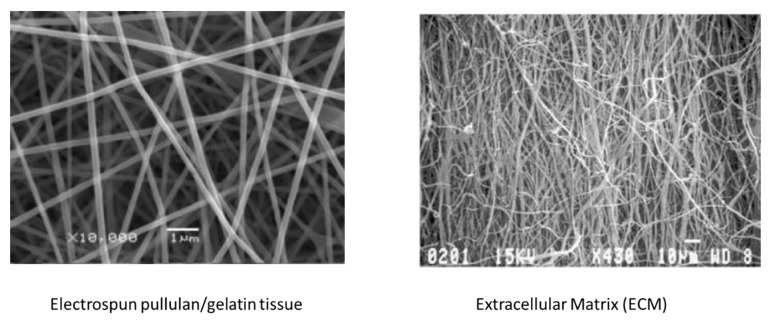
Comparison between electrospun pullulan/gelatin tissue [73] (**left**) with extracellular matrix [73] (**right**).

Skin is a biological barrier that is generally less than 2 mm thick, prevents water loss, and protects the body’s internal organs from the external environment and pathogen aggression (Figure 3) [74]. It is composed of two layers: the outer part, the epidermis, which is in contact with the environment, and the inner part, the dermis.

**Figure 4:**
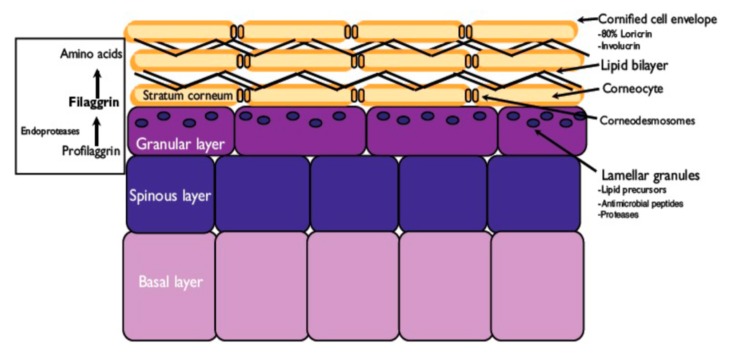
Production of amino acids and natural moisturizing factors (NMF) by filaggrin at the stratum corneum level.

The first layer, which can be further divided into the stratum corneum (SC), stratum granulosum, stratum spinosum, and stratum basalis, is composed of cellular layers of keratinocytes that self-renew their complex structure in 2–3 weeks. Keratinocytes, which produce keratin and make up more than 85% of the epithelial mass, are generated by mitosis in the basal layer of the epidermis. They pass outward through successive stages of differentiation to be shed at the skin surface together with a thin protective hydrolipidic film [75]. The dermis has a sponge-like structure composed of various ECM fibrous proteins and fibroblasts that synthesize collagen and elastin. While the skin outermost layer (i.e., stratum corneum made of corneocytes) prevents the skin delivery of any substance, the interior layers perform the task of keeping the skin hydrated and glossy. It is pointed out that keratinocytes, thanks to keratin, provide a covering tissue that is chemically unreactive, hard, waterproof, elastic, and resistant to any abrasion and physical insult. Moreover, these specialized cells secrete low-molecular weight mediators by which cells communicate and influence the activities of each other. On the other hand, the dead cell corneocytes, filled with keratin, water and enzymes, represent not only the main protective function as a barrier, but are also involved in the maintenance of skin flexibility and hydration by natural moisturizing factors (NMF), which is also necessary to retain water, reinforcing and supporting the collagen function [62,63]. NMF, which comprise between 20% and 30% of the corneocytes dry weight, is principally composed of amino acids or derivatives [i.e., pyrrolidine carboxylic acid (PCA)] together with lactic acid, urea, citrate and sugars [75]. Moreover, corneocytes are surrounded by a protein envelope layer linked to lipids (skin lamellae) connected with the lamellar bodies of the stratum granulosum that are rich in profilaggrin. The process that converts profilaggrin in filaggrin is fundamental for the organization and production of lipid lamellae and NMF (Figure 4). 

In any way, intercellular lipids and NMF are pivotal for the rate-limiting efficiency of the skin’s hydrophobic and hydrating barrier, forming the only continuous domain in the SC. The skin barrier function remains efficient when the presence of lipid lamellae is correctly balanced by an intact superficial hydrolipidic film enriched by NMF [75]. Thus, the necessity of formulating innovative cosmetic products that, thanks to efficient carriers and effective active ingredients, are able to pass through this barrier. As a result, the selected ingredients, released at the right skin layers, may have the possibility of communicating with the cells, with the aim to re-establish the intercellular communication, keep the stratum corneum hydrated, and renew the native ECM structure of the dermis when diseased or aged [76]. This activity requires the use of the right active ingredients and carriers such as pullulan and its composites, which are capable of regenerating the structural architecture not only of healthy skin, but also for wounded or aged skin when altered or destroyed by different injuries or diseases [77,78]. For instance, Li et al. [79] prepared hyaluronic acid grafted pullulan polymers and used them in the formation of novel biocompatible wound healing films. Moreover, Vora et al. [80] studied pullulan-based dissolving microneedle arrays for enhanced transdermal delivery of small and large biomolecules. In good agreement, the cosmetic industry is continually searching for new ingredients and functions in order to respond to the diversification of consumer needs. 

## 9. Pullulan and Personal Care/Cosmetic Market

Carriers play a critical role in the creation of a cosmetic product, helping to preserve the efficacy, safety, and stability of the selected active ingredients. They have to load, transport, and deliver the active ingredients at the programmed level of the skin to give the promised benefits to the customers. The detergent or cosmetic formulation is made of active ingredients and other compounds that form the physical structure of the vehicle/carrier that are necessary for delivering the “actives” at the level of different skin layers. These specialized compound-systems can affect the load and delivery of the active components by a number of different means [75]: interacting with the active agents, controlling the rate of their release from the carrier, altering the SC resistance, or enhancing its hydration state [77]. Optimal selection and use of the right carriers can assist cosmetic manufacturers in developing new and innovative products characterized for their enhanced functionality and cost-saving formulations. Due to increasing requests from consumers of nature-oriented products, natural polysaccharides and their derivatives represent a group of interesting polymers to be used as carriers to make the usual and classic emulsions as well as innovative cosmeceutical-tissues [22,23,24,38,39,40,41]. Specifically, they may be used to make not only the new micro/nano emulsions, but also the smart non-woven tissues made by natural fibers covered on their surface or bound into their structure by selected active ingredients [78,79]. Moreover, polysaccharides such as PUL, its derived compounds, and complexes may be used in the formulation and manufacture of new matrix systems, scaffolds, films micro/nano particles that resemble the natural ECM. Furthermore, as previously reported polysaccharides are not only biodegradable and toxicologically harmless, but also of low cost and relative abundance compared to their synthetic counterparts [59]. Thus PUL, given its particular physicochemical properties due to the multiple functional groups characterizing its biomolecules that are capable of different complex active compounds, can be processed into hydrogels, transparent, and edible films and tissues [78,79,80,81,82,83]. In addition, it shows an ideal capability to make advanced medications and innovative cosmetic products due to its safeness, effectiveness, water retention capacity, interesting gas barrier (i.e., the skin *perspiratio insensibilis*), and regenerative property to rejuvenate or reconstruct the ECM structural architecture of an aged or injured skin [76]. The aging process, in fact, slows down the skin mechanisms attributed to innate surveillance, reproduction, and cell growth, influencing their daily regeneration [82,83,84,85]. For this purpose, regenerative medicine aims to recuperate lost tissues by guiding cell growth and restoring the original tissue-scaffold architecture. Thus, the necessity of using biomaterials such as PUL or other polysaccharides, chitin nanofibrils, nanolignin, and their derivatives, as interesting and innovative polymers, is useful to reconstruct the aged skin structure [23].

On one hand, chitin nanofibrils, which keep balanced cellular metabolism and intercellular communication, seems able to stimulate collagen and elastin production and neutralize the oxidation phenomena caused by UV and pollution particles [22,23,24]. On the other hand, a composite consisting of polysaccharides containing chitin nanofibrils and its complex with lignin, mimicking the skin ECM structure, represents a biomimetic matrix that is able to simulate the effects of a natural tissue and favor cell growth and differentiation [39,40,41]. Scaffolds, in fact, as previously reported, should serve as a platform for cellular localization, adhesion, and differentiation as well as a guide for the development of new functional tissues. Therefore, the importance of the safe and effective reconstruction of these scaffolding-architectures, made by the incorporation of the right bioactive ingredients, is evident. They have to positively influence the skin cellular interactions via the creation of the same micro-nano, beneficial, biomimetic environmental structures, and should also be capable of stimulating biochemical communications [85]. Currently, the available cosmetic treatment options are lacking in establishing both functional and cosmetic satisfaction. Both women and men, are looking for effective and miraculous products because they do not want to appear aged and would like to obtain a durable juvenile aspect. For this purpose, and according to the Euromonitor International research study, the global and personal care market was worth US$ 454 billion in 2013, US$ 25 billion of which is represented by the anti-aging niche market [86,87,88]. Within this global market, 28% is represented by the Asia Pacific, achieving the highest value sales at US$ 128 billion in 2013, while the anti-aging market accounted for the largest share and the highest value growth of 76% from 2008 to 2013. This underlines that the aging population, projected to grow to ~1.5 billion in 2050, has become the major driver of the cosmetic market due to the strong desire to retain a youthful appearance, just as the younger population is looking to maintain their youthful look. For this reason, there appears to be a robust demand for anti-aging cosmetics in future years [89].

## 10. Final Remarks

Due to the interesting activity and effectiveness shown from PUL and polysaccharides, especially in the medical sector, it is necessary to evaluate them for a more intensive use of this polymer in applications related to personal care and cosmetics, not only as an innovative active ingredient, but also as a safe compound for biodegradable items and packaging. For all these reasons, the development of biocomposites produced using biopolymer matrices, natural fibers, and reinforcement materials from renewable resources such as polysaccharides and PUL, has become a current and extensive research area because of the recyclability and biodegradability of these raw materials. However, the final aim of nanoparticle/nanocomposite products for the skin has to enhance the permeation of the active ingredients through the skin, which is necessary to make an innovative smart product. The aim of the industry is to go toward a net positive and green direction with the goal to produce safe and effective products, thus reducing the environmental impact of the entire cosmetic supply chain from production to the distribution to the final consumer. Naturally, the major dream of the modern personal care and cosmetic industry is to have sufficient intellectual, scientific, and productive capacity for creating safe and effective products that are capable of reducing the appearance of fine lines, wrinkles, and black spots caused by the aging phenomena, while respecting the environment. To obtain these results, it is necessary to act at the level of the key biological target of senescence boosting and ameliorating the cutaneous architecture of SC and the dermis skin scaffold [90]. Targeting senescent skin, in fact, not only has a positive influence on the cell’s life, but also gives emotional benefits on the entire human body, ameliorating the quality of life. Last but not least the future personal care and cosmetic products must be packed in biodegradable containers to really respect the environment [91]. In-progress research studies are moving in this direction to obtain a deeper comprehension of the likeness and difference between the skin and natural polymer based supports, produced by both electrospinning and/or casting/filming technology. Innovative, biobased, and biodegradable tissues and films as well as soft and hard packaging, for their specific structure and the natural ingredients selected, may be capable of slowing down, partially or globally, the skin aging phenomena, and also in reducing or eliminating the great waste invading the planet. The amount of food lost or wasted every year is equivalent to approximately 1.3 billion tons/year [92,93], accounting for GHG emissions estimated at 3.3 Gtons [93]. Regarding plastic waste, it has been verified that the beauty industry annually produces around 120 billion units of packaging with a CAGR of 6%. It has been estimated, therefore, that in 2050, 12 billion tons of plastic in landfills will be generated from the cosmetic sector only [94]. Thus, for example, the use of products made by PUL, which are oil resistant, printable, and primarily all biodegradable and compostable, can help to improve the sustainability of waste management [95]. In conclusion, a more intensive production and use of PUL, obtained from agro-industrial waste [96] such as carob pod, molasses, grape skin pulp, starch, olive oil and so on, may be not only useful for many commercial applications, but also of great help in protecting the environment from pollution.
